# Comparative analysis of the vaginal bacteriome and virome in healthy women living in high-altitude and sea-level areas

**DOI:** 10.1186/s40001-023-01391-1

**Published:** 2024-03-07

**Authors:** Chaoran Li, Song jin, Oingbo Lv, Guangyang Wang, Yue Zhang, Shenghui Li, Wei zhang, Fang Long, Zhuowei Shen, Siqi Bai, Duoii Zhaxi, Fandou Kong, Qiulong Yan, Zhen Xiao

**Affiliations:** 1 Department of Obstetrics and Gynecology, First Affiliated Hospital, Dalian Medical University, Dalian, 116011 China; 2 Operating Room, First Affiliated Hospital, Dalian Medical University, Dalian, 116011 China; 3 Puensum Genetech Institute, Wuhan, 430076 China; 4 Department of Microbiology, College of Basic Medical Sciences, Dalian Medical University, Dalian, 116044 China; 5 Department of Obstetrics and Gynecology, People’s Hospital of Naqu, Naqu, Tibet 852000 China; 6 Institute of High Altitude Medicine, People’s Hospital of Naqu, Naqu, Tibet 852000 China

**Keywords:** Vaginal microbiome, Whole-metagenome shotgun sequencing, High-altitude area, Bacteriome, Virome

## Abstract

The vaginal microbiota plays an important role in the health of the female reproductive tract and is closely associated with various pregnancy outcomes and sexually transmitted diseases. Plenty of internal and external factors have strong influence on the changes in a woman's vaginal microbiome. However, the effect of a high-altitude on female vaginal microbiota has not been described. In this study, we characterized the vaginal bacteriome and virome of 13 and 34 healthy women living in high-altitude and sea-level areas, using whole-metagenome shotgun sequencing of their vaginal mucus samples. The results revealed that the vaginal bacteriomes of high-altitude individuals are featured by a significant increase of species diversity, depletion of *Lactobacillus crispatus*, and more abundant of some anaerobic bacteria, such as *Chlamydia trachomatis*, *Mageeibacillus indolicus*, *Dialister micraerophilus*, and *Sneathia amnii*). In addition, the vagina samples of sea-level subjects harbor more *Lactobacillus* strains, whereas the anaerobic bacteroidetes strains mostly appeared in high-altitude subjects. Identified and assembled 191 virus operational taxonomic units (vOTUs), there were significant differences in the abundance of 107 vOTUs between the two groups. Together, the results of this study raised the understanding of bacteriome and virome in the vagina of women at different elevations, and demonstrated that the vaginal microbiome is related to the high-altitude geographic adaptation.

**Supplementary Information:**

The online version contains supplementary material available at 10.1186/s40001-023-01391-1.

## Introduction

Numerous human cohort studies have provided insights into the role of human gut microbes in immune and metabolic functions [1, 2]. Based on consequence of these studies, we turned our attention to microbiome studies of parenteral mucosae, such as the vagina and oral. Microbiota studies of these sites have great potential for the treatment and prevention of related diseases. In general, the vaginal microbiome of healthy women is a less diverse community, with *Lactobacillus* as the dominant genus [3]. These microbial communities maintain normal vaginal pH and act as the first line of defense against the human immunodeficiency virus, bacterial vaginosis (BV) [4], and sexually transmitted infections [5, 6]. In addition, the vaginal microbiome also has been associated with several important reproductive outcomes, such as preterm birth [7], miscarriage [8], and infertility [9].Ravel et al. divided vaginal microbial composition and structure into five types of bacterial community state (CTS), four of which were dominated by *Lactobacillus* [10]. These CTS are adjusted by various factors, including the influence of ethnicity, physiological status of the vagina [11] and environmental factors [12], and are always in the process of dynamic change. Geographic variations are a key issue in microbiota-related studies [13], and this issue should not be ignored in vaginal microbiome studies. Differences in the geographical environment often represent differences in population genetic background, lifestyle, and dietary mode, and these factors drive the force of microbial community change. The frequency of each CST varies by race and geography [11, 13]. In Asian and white women, the vaginal microbiome is dominated by species of *Lactobacillus* (CSTI, II, III, and V) [3, 10]. Several studies have reported that most women of Asian descent have a vaginal microbiome with *Lactobacillus crispatus(L. crispatus)* as the main member, while *Lactobacillus iners* and *Gardnerella vaginalis* are the dominant microbiome in African women [3, 14, 15]. Yao et al. analyzed the characteristics of vaginal microbial in the populations of Henan, Guangdong, and Xinjiang province in China [16], and proposed that low abundance species-level OTU could distinguish populations in different regions. It means that regional factors have a strong influence on the variation of the female vaginal microbiome. Numerous studies have revealed the characteristics of vaginal bacterial and viral communities [17–20], but the research on the effects of different geographical locations on the vaginal microbiome of the Chinese native population is yet to be explored.

Qinghai–Tibet Plateau has a unique geographical environment. The climate at high altitude (an average of 4–5 km above sea level) poses various challenges to the survival of local inhabitants. Previous studies had demonstrated significant differences in gut microbiota between people living at high altitudes and those living at low altitudes [21]. Thus, this also led us to explore whether altitude adaptation may also affect the vaginal microbiome. In this study, we performed whole-metagenome shotgun sequencing and follow-up analyses on vaginal samples from 13 healthy inhabitants of high-altitude (Qinghai–Tibet plateau) area and 34 healthy inhabitants of low-altitude area (Dalian, a coastal city). We compared the structure and function of vaginal bacterial communities at the species and strain levels of individuals at high-altitude and sea-level areas, and also discussed the characteristics of vaginal viral communities. This study provides a basis for further understanding the effects of the biogeographic factor on female vaginal microbiota.

## Methods

### Subjects and vaginal sample collection

This study received approval from the ethics committee of People’s Hospital of Naqu (Tibet) and the First Affiliated Hospital of Dalian Medical University (Liaoning). Written informed consent was obtained from each participant. The whole process was carried out in accordance with the approved guidelines. In this study, all vaginal samples were collected from 37 and 53 healthy female residents from Naqu (~ 4,500 m above sea level in the middle of the Tibetan Plateau) and Dalian (a coastal city with ~ 20 m above sea level in the northeast of China), respectively. Due to the lower success rate of whole-metagenome shotgun sequencing of vaginal secretion samples, ultimately, only 13 high-altitude vaginal samples and 34 low-altitude vaginal samples were succeeded in the end. There were no significant differences in age or BMI between the two cohorts of subjects.

All recruited women were excluded from liver diseases, diabetes, hypertension, gastrointestinal disease, bacterial vaginosis, vulval and vaginal candidiasis, and trichomonas vaginitis. None of them took any antibiotic or microbial modulator within 3 months before sampling. The participants were asked for abstinence and avoiding vaginal douche at least 5 days before sample collecting. The vaginal secretion was collected from the posterior fornix of the participant’s vagina by trained gynecological doctors. The vaginal secretion samples were stored at −80 ℃ until further processing for experiments.

### DNA extraction and whole-metagenome shotgun sequencing

Metagenomic DNA was extracted from all vaginal secretion samples according to the manufacturer’s protocols provided in the TIANamp Stool DNA kit (Tiangen, China). Briefly, SA buffer (0.5 ml), SC buffer (0.1 ml), Proteinase K (0.015 ml), and 100 mg of zirconium beads were added to ~ 120 mg of sample and the pellets were homogenized and disrupted in 2 ml screw-cap tubes (Axygen) by a bead beater. The suspension was incubated at 95 ℃ for 10 min to lyse bacterial cells. After centrifugation (13,400 g, 3 min) and incubation of supernatant with RNase A (0.01 ml) and SH buffer on ice for 5 min, the supernatant after centrifugation (13,400 g, 3 min) was treated with an equal volume of GFA buffer. Then, the RNase-free spin columns were used to obtain total DNA of the solution. The extracted DNA was dissolved in 50 µl sterile water. The DNA concentration was quantified with NanoDrop2000. DNA quality was examined with a 1% agarose gel.

For each DNA sample, we constructed a 150 bp paired-end library with an insert size of approximately 350 bp. All libraries were barcoded and pooled to perform whole-metagenome shotgun sequencing at the Illumina NovaSeq platform. The raw sequencing reads of metagenomic data for each sample were independently processed for quality control using fastp (v.0.20.1) [22]. Reads ended with low-quality base (quality score < 30), ‘N’-containing or contaminated reads, and too short reads (< 90 bp) were removed based on the default parameters of fastp. The high-quality reads were then mapped to the human reference genome (GRCh38.p13) using Bowtie2 (v.2.4.1) [23], and the human sequences were removed from the data.

### Analyses of the vaginal bacteriome

The species taxonomic composition generated in metagenomic data of vaginal samples was implemented using the MetaPhlAn3 (v.3.0.7) [24] algorithm. Of the results obtained, only taxa belonging to the bacteria domain were included in subsequent vaginal bacteriome analysis. The HUMAnN3 (v.3.3.2) [24] algorithm was used to obtain the functional composition from the vaginal metagenomes, including the abundance spectrum of microbial metabolic pathways and molecular functions. The diversity indexes (Shannon and Simpson) at the species and MetaCyc pathway levels were calculated based on the relative abundance spectrum of species, using the *vegan* (v.2.5–6) package of the R (v.4.0.3) platform. The rarefaction curve was calculated based on MetaPhlAn3 results and plotted using the ggpubr (v.0.4.0) package of the R platform.

The methodology of prokaryotic metagenome-assembled genomes (MAGs) had been reported in several studies [2, 25, 26], and we analyzed vaginal metagenomic data using assembly procedures referring to these standards. In the workflow, MEGAHIT (v.1.2.9) [27] was used for metagenomic assembly of high-quality clean reads. To obtain more accurate assembly results, we selected a wider range of k-mer sizes (21, 41, 61, 81, 101, 121, and 141 bp), which were considered to be effective in improving the quality of assembly results [28]; The assembled contigs (> 2kbp) were binned implemented using MetaBAT2 (v.2.15) [29] with default parameters; all bins were evaluated for quality using the *lineage_wf* workflow in CheckM (v.1.1.3) [30], and the bins with greater than 50% integrity and less than 10% contamination were filtered for further analysis. The taxonomic classification of the MAGs was implemented based on the Genome Taxonomy Database [31] using the workflow in GTDB-tk (v.1.4.0). According to the prokaryotic species classification definition criteria [32], genomes with an average nucleotide identity (ANI) greater than 95% were considered to be the same species. Based on this standard, we used dRep (v.2.2.3) [33] to cluster MAGs at the species level.

### Identification of viral sequences and analyses of the vaginal virome

We followed the methodology developed by several recent studies to identify viral sequences from vagina metagenomic data [34, 35]. Only metagenome-assembled contigs with lengths larger than 5 kbp could be included in virus prediction and analyses. In this study, we used three approaches for prediction, respectively: (1) in CheckV (v.0.7.0) [36] evaluation results, the number of viral genes is greater than the number of bacterial genes; (2) the results of VIBRANT (v.1.2.1) [37], and (3) the result of DeepVirFinder (v.1.0) [38] with score > 0.9 and *p* < 0.01. Contigs that were predicted to be viruses in the above methods were collected and evaluated for quality using CheckV. Finally, BLASTN (v.2.11.0 +) [39] was used to remove redundancy in these viral contigs with 95% nucleotide similarity and 70% sequence coverage as thresholds to obtain the catalog of viral operational taxonomic units (vOTUs).

The human gut virus databases were downloaded from publicly available resources, including Gut Virome Database (GVD) [35], Gut Phage Database (GPD) [40], and Metagenomic Gut Virus catalog (MGV) [41]. BLASTN was used to look for the sharing of these virus databases with vOTUs in this study. Virus sequences with sequences similarity greater than 95% and coverage greater than 75% were considered to be of the same species. Protein sequence prediction of vOTUs was achieved using Prodigal (v2.6.3) [42] with option ‘-meta’. The taxonomical classification of vOTUs was done through an adapted vConTACT2 (v.2.0) pipeline [43]. The pipeline provided a protein database, including vConTACT2 reference, protein of *crAss-like phages* [44], and viral protein from Benler et al.’s study [45]. DIAMOND (v.2.0.6.144) [46] algorithm with options “–query-cover 50 –identity 30 –top 40 –score 50” was used to align protein-coding genes of vOTUs and protein database. A virus was considered to belong to a known family when 25% of its genes are assigned to that family. To predict the potential bacterial host of the vOTUs, CRISPR Spacers in 76 MAGs sequences were predicted using MinCED with parameter ‘-minNR 2’, and these fragments were aligned to vOTU sequences using BLASTN (blastn-short mode) to determine virus–host matches. The bacteria that matched the most spacer sites were considered the primary host. Analysis of the functional composition of vOTUs was performed based on the KEGG (Kyoto Encyclopedia of Genes and Genomes) database [47], and DIAMOND was also used to complete the matching task of amino acid sequence. The hit with coverage greater than 50 and a score greater than 60 was considered effective, and each gene was assigned to the optimal hit.

### Correlation network

The SparCC algorithm [48] was used to calculate the correlation coefficient between bacterial species and vOTUs based on their relative abundance spectrums. Only strong inter-correlations (*r* > 0.6 or < −0.6) were retained to clearly show associations between species. Visualization of the correlation network was achieved by Cytoscape.

### Statistical analysis

Statistical analyses involved in this study were implemented on the R 4.0.3 platform. Principal coordinates analysis (PCoA) was implemented based on the Bray–Curtis distance at the bacterial species and vOTU levels, using the *vegan* package. Permutational multivariate analysis of variance (PERMANOVA) was performed by the *adonis* function in the *vegan* package, where *p* values were generated based on 1,000 permutations. Wilcoxon rank-sum test was used to compare the differences in microbial diversity and taxonomic profiles between the two cohorts. The Benjamini–Hochberg procedure was used to perform multiple corrections to convert the *p* values to *q* values.

### Data availability

The raw whole-metagenomic shotgun sequencing data, sample metadata, and statistical scripts acquired in this study are available from the corresponding author on reasonable request. The raw whole-metagenome shotgun sequencing data used in the study have been deposited in the European Nucleotide Archive (ENA) at EMBL–EBI under accession number PRJEB51898 (https://www.ebi.ac.uk/ena/browser/view/PRJEB51898).

## Results

### Biodiversity, phylogenetic and functional composition of the vagina bacteriome

**Fig. 1:**
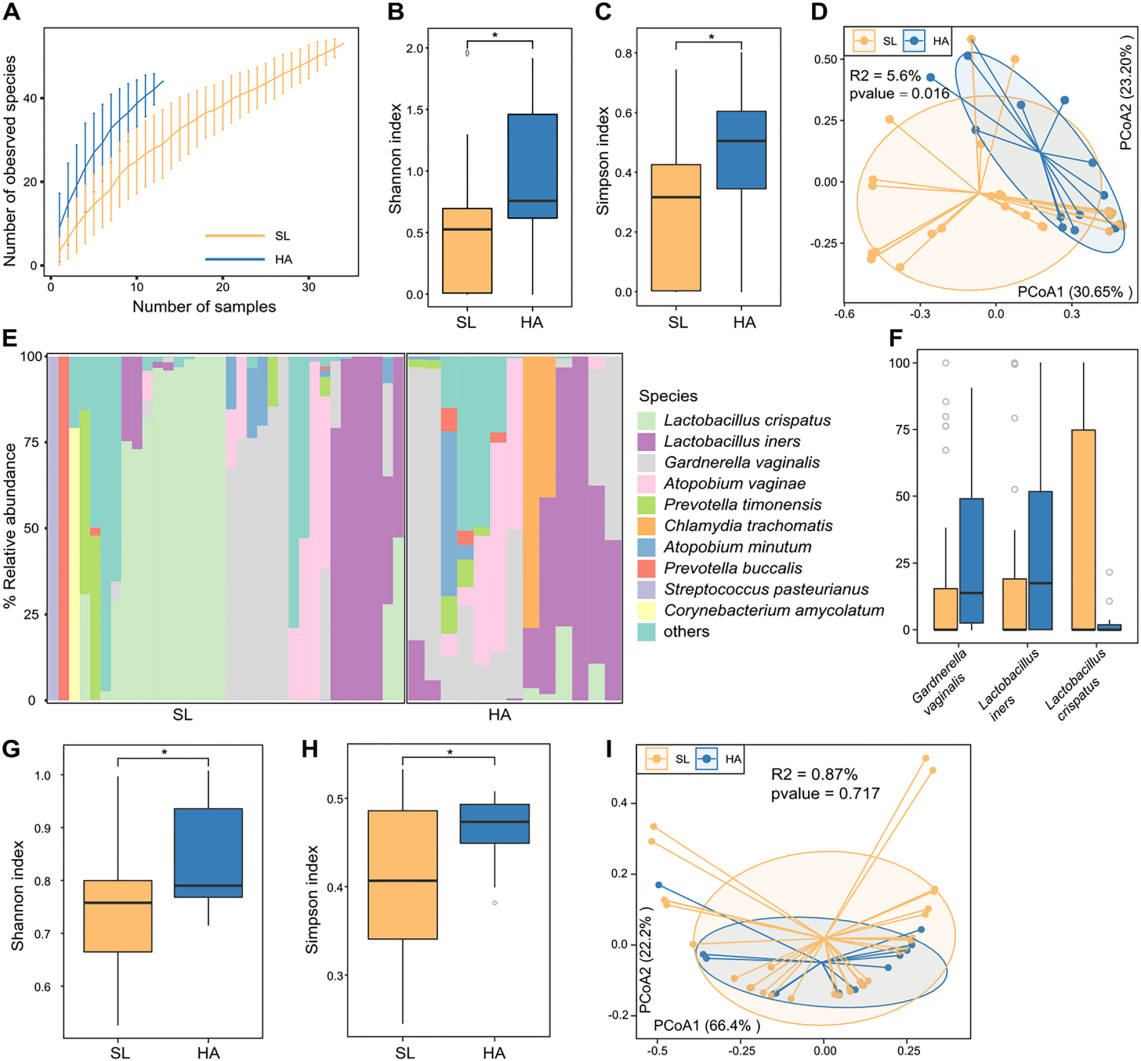
Difference in vaginal bacteriome between HA and SL subjects. **A** Rarefaction curve analysis of the number of observed species on HA and SL group. The number of species in two groups is calculated based on a randomly selected specific number of samples with 30 replacements, and the whisker line shows the median and quartile. **B**, **C** Boxplot shows the Shannon diversity index (**b**) and the Simpson index (**c**) of vaginal bacteriome between HA and SL groups. The significance level in the Wilcoxon rank-sum test is denoted as: *p < 0.05. **D** PCoA analysis of Bray–Curtis distance based on the vaginal bacteriome. Locations of samples in the first two principal coordinates are shown, and the sample points in the same group are linked by lines, and ellipses cover each group of samples near the center of gravity. **E** Composition of vaginal bacteriome at the species level. **F** Boxplot shows the relative abundances of three representative differential vaginal species in the HA and SL individuals. **G**, **H** Boxplot shows the Simpson index (**g**) and Shannon diversity index (**h**) of vaginal functional composition that differs between the two groups. The significance level in the Wilcoxon rank-sum test is denoted as: *p < 0.05. **I** PCoA analysis of Bray–Curtis distance based on the vaginal functional composition

Based on whole-metagenome shotgun sequencing, we obtained total 273.2 Gbp high-quality data from the vaginal samples of 34 sea-level (SL) and 13 high-altitude (HA) urban residents. The total 23.4 Gbp non-human data were obtained after removing human genome sequence contamination (average 0.5 Gbp per sample; Table S1). To investigate the composition of SL and HA subjects’ reproductive vaginal bacteriome, we analyzed the metagenomic data using MetaPhlAn3 [24]. A total of 184 taxa were obtained from all samples, including 9 phyla, 15 classes, 19 orders, 29 families, 39 genera, and 73 species. Rarefaction analysis showed that the number of species observed in HA subjects was higher than that of SL subjects with the same sample size (Fig. 1A), though the curve was unsaturated. The Shannon and Simpson indexes at the species level of the HA group were higher than that of the SL group (*p* < 0.05) (Fig. 1B, C). These results suggested that high altitudes people have a more complex vaginal bacteriome than usual expected. Next, principal coordinate analysis (PCoA) of Bray–Curtis distance was undertaken to further understand the differences of vagina bacteriome between HA and SL individuals. Remarkable alteration between the HA and SL groups (PERMANOVA *R*^2^ = 5.6%, *p* = 0.016; Fig. 1D) suggested the significant differences in overall vaginal bacteriome structure between HA and SL.

At the species level, we found significant individual variability in the vaginal bacteriome of each subject in both HA and SL groups (Fig. 1E). Overall, the vaginal bacteriome of SL subjects was mainly composed of *L. crispatus* (average relative abundance 31.1%), *Lactobacillus iners* (18.4%), and *Gardnerella vaginalis* (17.1%), while that of HA subjects was dominated by *Gardnerella vaginalis* (28.7%), *Lactobacillus iners* (28.6%), *Atopobium vaginae* (12.6%), and *Chlamydia trachomatis* (9.2%). *L. crispatus* showed a significant depletion in the vaginal bacteriome of HA subjects compared with that of SL subjects, even though statistical tests were not significant (average relative abundance 2.9% vs. 31.1%, *p* = 0.054), whereas *Lactobacillus iners* and *Gardnerella vaginalis* were significantly more abundant in HA subjects (Fig. 1F, *p* < 0.05 for both). Except these, 10 bacterial species had significantly differed in relative abundances between two cohorts (Table S2; *p* < 0.05), and all of them were enriched in HA subjects, including some anaerobic bacteria, such as *Chlamydia trachomatis*, *Mageeibacillus indolicus*, *Dialister micraerophilus*, and *Sneathia amnii*.

Then, we analyzed the vaginal metagenomic data of HA and SL groups from the functional perspective. A total of 342 MetaCyc pathways were predicted for comparative analysis between HA and SL subjects (Table S3). Similar to the pattern at the species level, the Shannon and Simpson indexes of vaginal functional composition in the HA group were significantly higher than those in the SL group (Fig. 1G, H). However, PCoA analysis at the functional level showed no significant difference in functional composition between the two groups (PERMANOVA R^2^ = 0.87%, *p* = 0.717; Fig. 1I). This meant that their difference is relatively small, but the Wilcoxon rank-sum test found significant differences in 8 of 342 MetaCyc pathways between the HA (5 enriched) and SL (3 enriched) groups (Table S3). The HA-enriched pathways included methylerythritol phosphate pathway I (NONMEVIPP–PWY), 1,4-dihydroxy-6-naphthoate biosynthesis II (PWY-7371), L-histidine degradation III (PWY-5030), thiamine diphosphate salvage II (PWY-6897), and glycogen biosynthesis I (GLYCOGENSYNTH–PWY), while the SL-enriched pathways included tetrapyrrole biosynthesis I (PWY-5188), petroselinate biosynthesis (PWY-5367), and superpathway of L-aspartate and L-asparagine biosynthesis (ASPASN–PWY).

### Strain-level comparison of the vaginal bacteriome

**Fig. 2:**
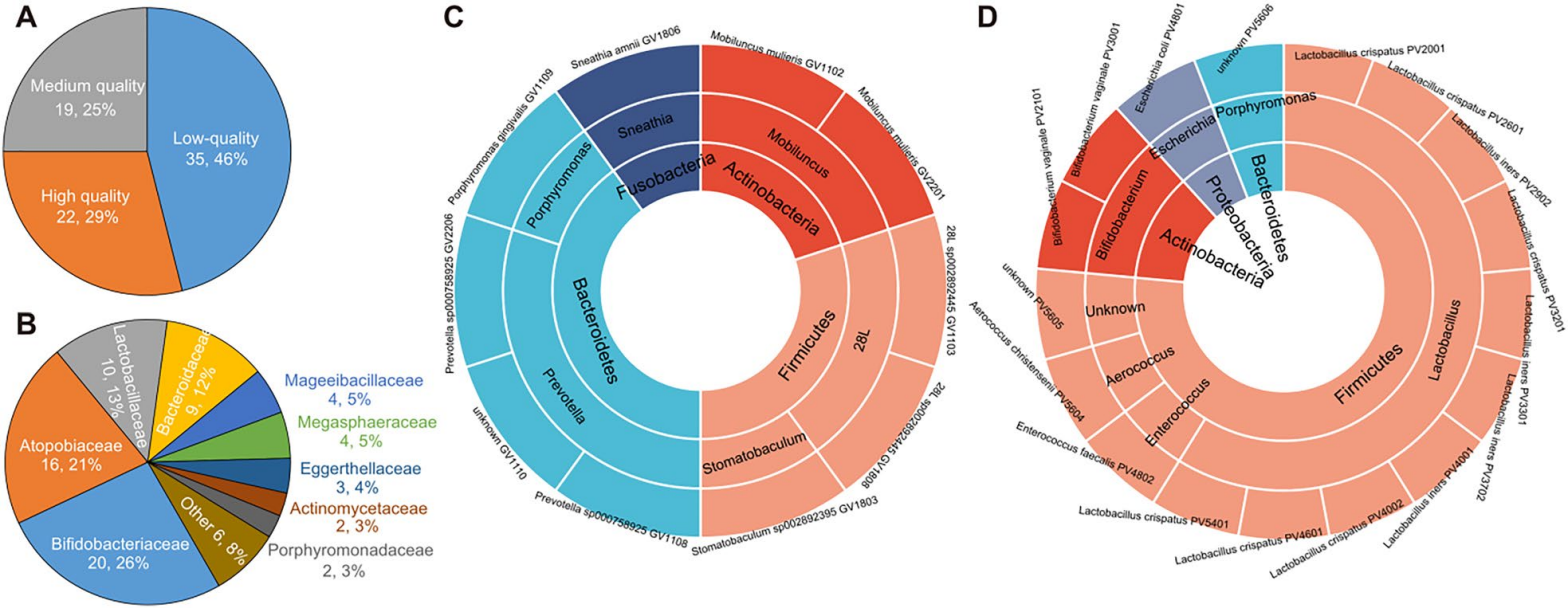
Comparison of metagenome-assembled genomes between HA and SL subjects. **A** Pie plot shows the proportions of high-, medium- and low-quality MAGs constructed by this study. **B** Pie plot showed the proportions of bacterial family-level assignment of the MAGs. **C**, **D** Taxonomic distribution of the HA-specific (**c**) and SL-specific (**d**) MAGs. For each sunburst chart, the stains names of MAGs are shown at the outermost circle, and the genus- and phylum-level taxonomic information are shown at the inner ring

Next, we analyzed the genomes of vaginal bacteriome in plateau and plain populations at the strain level. A total of 76 metagenome-assembled genomes (MAGs) were reconstructed from 30 high depth sequenced vaginal samples (8 HA samples and 22 SL samples; Table S4). According to minimum information about a metagenome-assembled genome (MIMAG) [49] standard of bacteria, 29% (22/76) MAGs reached high quality (completeness > 90% and contamination < 5%), and the remaining was medium-quality (completeness 70–90%, contamination < 5%; *n* = 19) and low-quality (completeness 50–70%, contamination < 5%; *n* = 35) genomes (Fig. 2A). The majority of these MAGs were members of Actinobacteria (*n* = 41, mainly composed of Bifidobacteriaceae and Atopobiaceae members), followed by Firmicutes (*n* = 22) and Bacteroidetes (*n* = 11) (Fig. 2D). In addition, the remaining were only two MAGs, *Escherichia coli* (belonged to Proteobacteria) and *Sneathia amnii* (belonged to Fusobacteriota). We clustered the MAGs base on an average nucleotide identity (ANI) threshold of 95% for prokaryotic species definition [32] and compared their presence in the HA and SL groups. Based on 76 MAGs, 27 species-level clusters (referred to as species hereinafter) were obtained. Of these, 7 species (representing 10 MAGs) were only concentrated in the vaginal bacteriome of HA subjects, whereas 8 species (representing 17 MAGs) were uniquely assembled from the SL subjects (Fig. 2C, D; Table S4). The HA-specific species mainly belonged to Bacteroidetes, including *Prevotella sp000758925*, *Porphyromonas gingivalis*, and an unclassified *Prevotella* spp., and the rest were *Megasphaeraceae-28L sp002892445*, *Stomatobaculum sp002892395*, *Mobiluncus mulieris*, and *Sneathia amnii(S. amnii)*. It is noteworthy that *S. amnii* is an opportunistic pathogen and may cause infections during pregnancy or in the post-partum period [7, 50]. On the other side, 8 SL-specific species included 5 Firmicutes members, which are *Aerococcus christensenii*, *Enterococcus faecalis*, *Lactobacillus crispatus*, *Lactobacillus iners*, *Bifidobacterium vaginale*, *Escherichia coli*, and unclassified *Porphyromonas* spp. and Fastidiosipilaceae species. *Bifidobacterium vaginale* is a closely related bacterium to bacterial vaginitis [51], which need more functional verification in our future research. Taken together, these results suggest that the presence of certain bacterial species are closely related to the geographic location of the vaginal bacteriome of healthy women.

### Construction of vaginal viral catalog and comparison of the vaginal virome

**Fig. 3:**
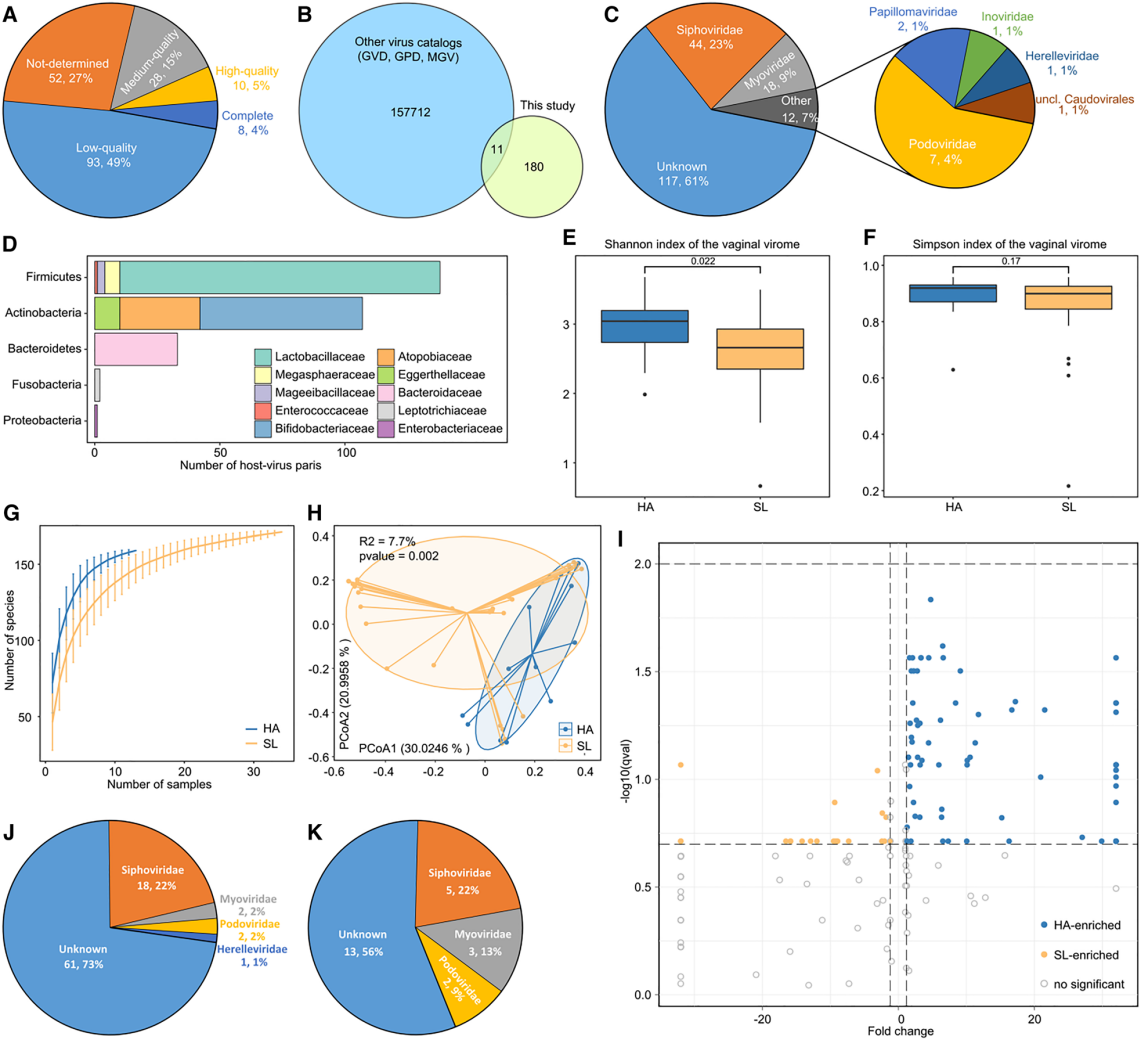
Characteristics of the vaginal virome. **A** Pie plot shows the proportion of vOTUs of different qualities in the non-redundant virus catalog. **B** Venn plot shows the overlap of the virus catalog and the other three virus genome databases. **C** Pie of pie chart shows the family-level taxonomic annotation of the vOTUs. **D** Bacterial host assignment of the vOTUs. **E**, **F** Boxplot shows the Shannon diversity index (**f**) and the Simpson index (**g**) of vagina virome among two groups. The significance level was assessed using Wilcoxon rank-sum test. **G** Rarefaction curve analysis of the number of observed vOTUs on two groups. **H** PCoA analysis of Bray–Curtis distance based on the composition of vaginal virome. Shows the locations of samples in the first two principal coordinates, the sample points in the same group are linked by lines, and ellipses cover each group of samples near the center of gravity. **I** Volcano plot shows the fold change (*X*-axis) and *q* values (-log10 transformed; *Y*-axis) for all vOTUs. The vOTUs that enriched in HA and SL subjects (*p* < 0.05) are shown in blue and yellow dots, respectively. **J**, **K** Pie plots show the taxonomic distribution of HA-enriched vOTUs (**j**) and SL-enriched vOTUs (**k**)

We next focused on the characteristics of the virome in the vaginal samples of HA and SL individuals. We identified 1,162 highly credible viral sequences greater than 5,000 bp in length from metagenomic data of 47 vaginal samples. After removing redundant sequences at 95% nucleotide similarity, a total of 191 viral operational taxonomic units (vOTUs) were obtained (Table S5). The vOTUs length ranges from 5,443 bp to 159,420 bp, with an average length of 24,674 bp and N50 length of 31,883 bp. According to CheckV evaluation results of completeness (Fig. 3A), 4% (8/191) of vOTUs were complete viral genomes, while 5% (10/191) and 15% (28/191) of vOTUs were high and medium quality viral genomes, respectively. In addition, we checked with the currently available collections of human gut virome (i.e., GVD, GPD, and MGV) and found that only 11 vOTU had been previously assembled in these existing databases (Fig. 3B). This result suggests that the shared range between vaginal and gut viral populations is very small, which may be related to the huge environmental differences between them. The vast majority of vOTUs (61.3%) could not be assigned to a known viral family (Fig. 3C), whereas 23.0% of vOTUs belonged to the Siphoviridae family and 9.4% of vOTUs belonged to the Myoviridae family, and the remaining 6.3% of vOTUs were from Podoviridae (3.7%), Papillomaviridae (1.0%), Inoviridae (0.5%), Herelleviridae (0.5%) and unclassified Caudovirales (0.5%) members. Subsequently, 53.9% of vOTUs could be assigned to at least one bacterial host based on their homology to 76 MAGs or CRISPR spacer similarity of these genomes. Lactobacillaceae, Bifidobacteriaceae, Atopobiaceae, and Bacteroidaceae members were the most common viral hosts (Fig. 3D; Table S5).

To investigate the vaginal virome signatures in HA and SL individuals, we assessed the diversity of viromes in two cohorts. Compared with SL subjects, the Shannon index of the vaginal virome in HA subjects was higher (*p* = 0.022) (Fig. 3E), while the Simpson index was not significantly differed between them (*p* = 0.17) (Fig. 3F). For the HA and SL groups, rarefaction analysis shows that the accumulative curve is close to saturation near 10 samples (Fig. 3G). Under the same sample size, the number of vOTU observed in HA subjects was larger than that in SL subjects. Similarly, PCoA analysis at vOTU level captured significant separation (PERMANOVA R^2^ = 7.7%, *p* = 0.002) between the HA and SL cohorts (Fig. 3H). Furthermore, we compared the vaginal viral profiles between HA and SL subjects at the vOTU level. The relative abundances of 107 vOTUs were significantly different between the two groups (*p* < 0.05 and fold-change > 1.2; Fig. 3I), including 84 HA-enriched and 23 SL-enriched vOTUs. The HA-enriched vOTUs included 18 Siphoviridae members and 5 viruses belonged to Podoviridae, Myoviridae, and Herelleviridae, while the SL-enriched vOTUs contained 4 Siphoviridae, 3 Myoviridae, and 2 Podoviridae members (Fig. J–K).

We predicted 1,931 genes from these 107 differential vOTUs. Based on KEGG database, 27.0% of genes were assigned to 322 KOs (Table S6). Due to the small amount of enriched vOTUs in the SL group, we could not observe the significant difference between the two cohorts. Nonetheless, we found that in HA-enriched vOTU, lysozyme (K07273), single-strand DNA-binding protein (K03111), anti-repressor protein (K07741), phage terminase large subunit (K06909), and site-specific DNA recombinase (K06400) were encoded at higher frequencies. These enzymes are involved in signaling and cellular processes or genetic information processing and may be associated with higher viral diversity in the vaginal ecosystems of HA subjects.

### Correlation network between vaginal bacteriome and virome

**Fig. 4:**
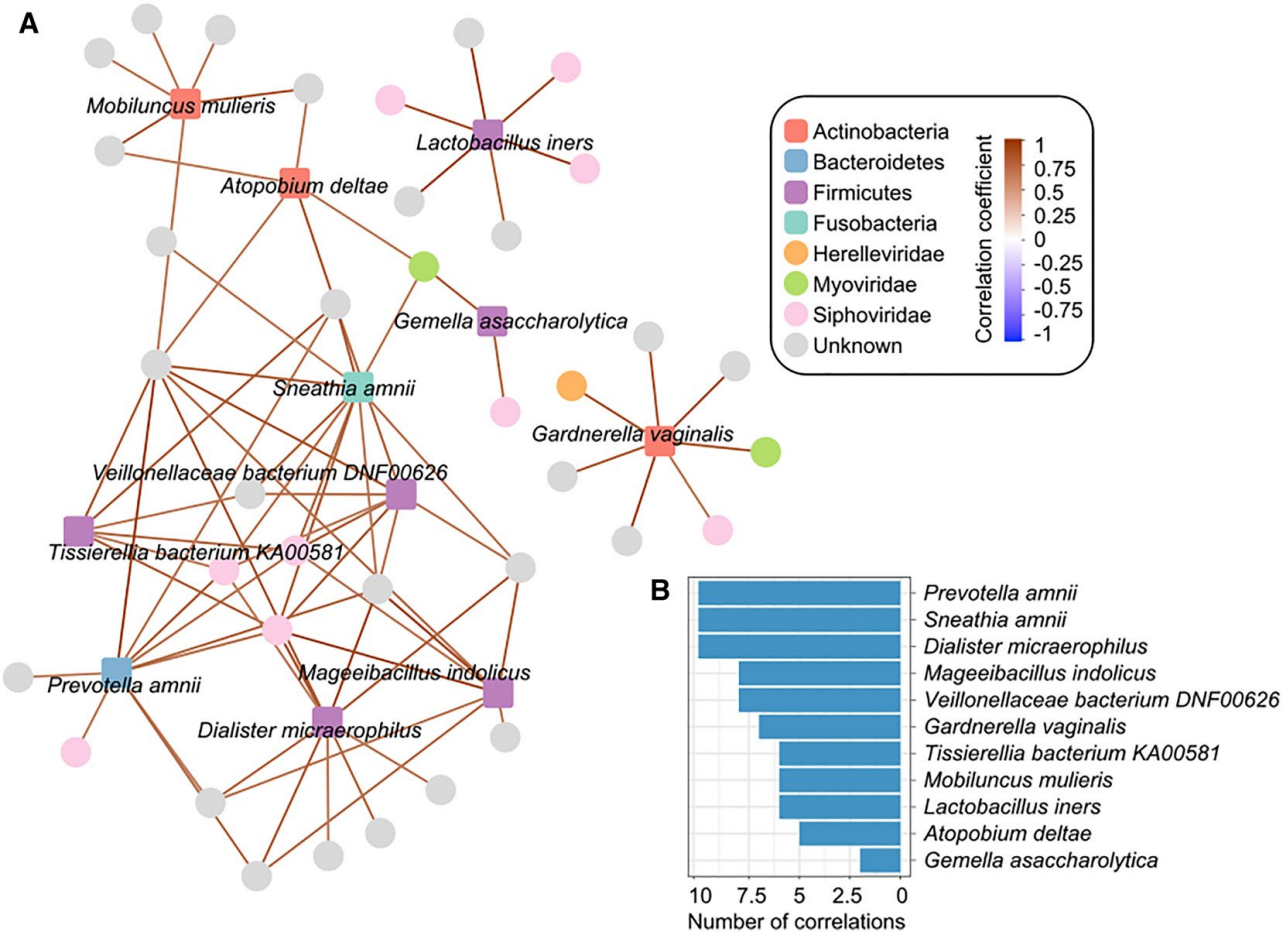
Correlation analysis between the bacterial species and viruses. **A** Correlation network between 12 bacterial species and 62 vOTUs. Square nodes depict the bacterial species (color fill according to their phylum-level taxonomic assignment), and circle nodes depict the vOTUs (color fill according to their family-level taxonomic assignment). Connecting lines represent the SparCC correlations with graduated colors: brownish red, correlation coefficient > 0.6; blue, correlation coefficient < −0.6. **B** Bar-plot shows the number of correlations of 12 bacterial species

To explore the relationship between vaginal bacteria and vaginal viruses, we performed a correlation analysis on 12 differential bacterial species and 107 differential vOTUs using the SparCC algorithm. We observed 78 virus–bacterium pairs of strong correlations between 11 bacteria and 37 viruses (correlation coefficient > 0.6 or < −0.6; Fig. 4A; Table S7). Several bacterial species, such as *Prevotella amnii*, *Dialister micraerophilus*, and *Sneathia amnii*, were more abundant in the vaginal bacteriome of HA cohort which were positively correlated with various viruses (Fig. 4B). These findings suggest that the virus–bacterium interaction network in the vaginal microbial community of HA subjects is vastly different from that of SL inhabitants.

## Discussion

High altitude areas attract the attention of researchers which have some unique geographical characteristics, such as low air pressure, low oxygen concentration, high UV exposure, and low annual average temperature. Meanwhile, the living conditions of the plateau are more severe [52], such as low boiling point, lack of urban running water systems, and lack of fresh fruits and vegetables. All of these plateau-specific factors may indirectly contribute to the diversity of human microbiota. Accordingly, gut microbiome differences between inhabitants of high-altitude and low-altitude areas were assessed earlier [53]. However, as a part of the reproductive tract system closely related to the microbiota, the effect of altitude adaptation on the structural and functional characteristics of bacteriome and virome of vagina has not been reported. We designed a study that explored the impacts of the geographical variations on the microbiome (bacteriome and virome) in the vaginal mucosa of female subjects. Herein, we collected 37 HA vaginal samples and 53 SL vaginal samples. Due to the insufficient sample dry matter and the nature of vaginal secretions, we observed a lower success rate. Finally, the bacteriome and virome characteristics of vaginal samples from HA (*n* = 13) and SL (*n* = 34) inhabitants were successfully analyzed using whole-metagenome shotgun sequencing. Like the results of gut microbiome research [52, 54], our results suggested that the distinguishably bacterial and viral communities existed in the vagina of HA and SL inhabitants. In addition, the HA inhabitants had a higher species and functional diversity of vaginal bacteriome and virome than SL inhabitants. Currently, there is no consensus as to whether high altitude is a factor in the altered vaginal microbiome, but in any case, the unique vaginal microbiome partly explains the specific effects of the living environment factors on a healthy vaginal ecosystem.

Enriched *Lactobacillus iners* and *Gardnerella vaginalis* but less *L. crispatus* were found in the HA subjects comparing with that of SL subjects. It is well-known that the characteristics of vaginal microbiota in healthy women is generally dominated by *Lactobacillus* genus bacteria and low species diversity. Studies have shown that women with a vaginal microbiome dominated by *Lactobacillus crispatus* have a fivefold lower risk of developing BV than women's vagina predominantly colonized by other bacteria [55]. In addition, enriched *Lactobacillus iners* and *Gardnerella vaginalis* were commonly found in BV patients [15]. The vaginal microbiome characteristics of the HA subjects were similar to those of BV, such as depletion of *Lactobacillus crispatus*, overgrowth of *Gardnerella vaginalis* and *Prevotella* spp. Therefore, vaginal bacterial factors may be related to the fact that a higher risk of vaginitis in the highland population. Coincidentally, African American and sub-Saharan African women were more likely to be colonized with BV-related bacteria, such as *Lactobacillus iners* or *Gardnerella vaginalis* [56]. Thus, this similarity in different geographical situations, cultural practices, and genetic factors suggested that unknown unanimous effects may promote the growth of these bacteria. In addition, proven or potentially vaginal pathogens, such as *Dialister micraerophilus* [57], *Sneathia amnii* [58], were also observed enriched in the vaginas of HA subjects. The strain level comparison showed that most strains of HA group concentrated in Bacteroidetes, while the more strains in the SL group belonged to *Lactobacillus* species. These findings suggested that we should be aware of the risk of vaginal inflammation induced by vaginal microbiota in women living in high altitude areas, even though they may not have obvious clinical symptoms.

As to virome, compared with SL subjects, HA subjects have a more complex and diverse virome in their vaginas, which may be related to their higher diversity of bacteriome. Anaerobic bacteria, such as *Prevotella amnii* and *Dialister micraerophilus*, may contribute to the enrichment of associated viruses in the vagina [59], and most of these viruses are unidentified species. In another study on pregnant women, higher vaginal viral diversity was thought to be associated with preterm birth, and changes in viral diversity are positively correlated with variation of bacterial diversity [60]. Jakobsen et al. reported that the vaginal virome was clearly linked with BV [18]. These prompted us to ask the question: does altitude adaptation of virome contribute to a higher risk of vaginitis? Unfortunately, due to the small sample size and lack of longitudinal sampling, we are unable to further interpret these results. Assessment of the species composition, functional characteristics, and impact on the reproductive health of the vaginal microbiome (bacteria, viruses, and fungi) is critical. Larger longitudinal clinical cohort or animal model trials are needed in the future to assess the causal relationship between the microbiome and geographic adaptation, with full consideration of individual confounding factors (diet, hygiene, and genetic background).

In summary, we systematically described the characteristics of the vaginal microbiome of individuals from 34 low-altitude and 13 high-altitude areas, and demonstrated that altitude adaptation contributed significantly to the changes of vaginal bacteriome and virome. We reveal functional characteristics between the two populations, as well as differences in strain levels, that will advance the understanding of high-altitude environments for health-related microbes of the vagina. One concern we raise from these differences is the negative impact of altitude environments on women's vaginal health. At present, there is insufficient evidence to explain the physiological mechanisms behind these phenomena. Future studies must further understand the causal relationship between the vaginal microbiome and the geographic factors, which can help design-specific treatment regimens for different geographical populations.

### Supplementary Information

**Additional file 1:** Supplementary tables.

## Data Availability

The raw whole-metagenomic shotgun sequencing data, sample metadata, and statistical scripts acquired in this study are available from the corresponding author on reasonable request. The raw whole-metagenome shotgun sequencing data used in the study have been deposited in the European Nucleotide Archive (ENA) at EMBL–EBI under accession number PRJEB51898 (https://www.ebi.ac.uk/ena/browser/view/PRJEB51898). The data sets used and/or analyzed during the current study are available from the corresponding author on reasonable request.
